# Benzo[*a*]pyrene induces NLRP1 expression and promotes prolonged inflammasome signaling

**DOI:** 10.3389/fimmu.2023.1154857

**Published:** 2023-05-04

**Authors:** Risa Kohno, Yuka Nagata, Tomohiro Ishihara, Chisato Amma, Yayoi Inomata, Takafumi Seto, Ryo Suzuki

**Affiliations:** ^1^ Laboratory of Hygienic Chemistry, Faculty of Pharmaceutical Sciences, Institute of Medical, Pharmaceutical and Health Sciences, Kanazawa University, Kanazawa, Japan; ^2^ Institute of Nature and Environmental Technology, Kanazawa University, Kanazawa, Japan; ^3^ Faculty of Frontier Engineering, Institute of Science and Engineering, Kanazawa University, Kanazawa, Japan

**Keywords:** airborne pollutants, benzo[a]pyrene, lung epithelium, inflammasome, NLRP1, aryl hydrocarbon receptor, reactive oxygen species

## Abstract

Benzo[*a*]pyrene (BaP), a polycyclic aromatic hydrocarbon in the air, triggers pulmonary inflammation. This study focused on BaP-induced inflammation in the alveolar epithelium. A549 cells were stimulated with BaP for four days. BaP treatment markedly increased NLRP1 expression but slightly decreased NLRP3. Furthermore, aryl hydrocarbon receptor (AhR) knockdown displayed no increase in BaP-induced NLRP1 expression. Similar results were also observed by blocking reactive oxygen species (ROS), which is mediated through AhR, suggesting that the AhR-ROS axis operates in BaP-induced NLRP1 expression. p53 involvement in ROS-mediated NLRP1 induction has also been implied. When we confirmed inflammasome activation in cells treated with BaP for four days, while BaP transiently activated NLRP3, it predominantly activated the NLRP1 inflammasome. These findings have led to the conclusion that BaP could be a potential ligand for the NLRP1 inflammasome persistently observed in the lung epithelium. Our study may provide additional evidence for the sustained pulmonary inflammation caused by environmental air pollution.

## 1 Introduction

Environmental air pollution is an emerging global issue that significantly impacts lung homeostasis and inflammatory responses. Ambient fine particulate matter with a diameter of up to 2.5 μm (PM_2.5_) is a widespread environmental contaminant with immunotoxic and carcinogenic potential (1). The size of particles (such as PM_2.5_ and PM_10_) is potentially associated with health issues because small particles reach deep into the lung tissues. Earlier studies have emphasized the pro-inflammatory action of PM_2.5_, which triggers a series of biological reactions or enhances lung inflammation (2, 3). Furthermore, PM_2.5_ is also involved in cardiovascular diseases, intrauterine growth restriction, and respiratory/pulmonary diseases (4–7). PM_2.5_ is a mixture of several harmful substances, such as organic and inorganic chemicals (3). Identifying its specific health-relevant features is of significant interest.

Polycyclic aromatic hydrocarbons (PAHs) are the primary organic components in PM_2.5_ formed during incomplete combustion or pyrolysis of organic matter. The toxicity and hazardous effects of PAHs have long been recognized as a public health concern in industrial and urban locations. Among various PAHs, benzo[*a*]pyrene (BaP), accounting for 27%–67% of airborne particle toxicity (8), is the most extensively investigated compound. BaP can induce direct DNA damage and mutations in growth-controlling genes, such as tumor suppressors or oncogenes. Therefore, it was classified as a Group 1 human carcinogen by the International Agency for Research on Cancer. Other PAHs have been ranked according to their carcinogenic potency relative to BaP using toxic equivalence factors. Additionally, BaP can cause lung inflammation and induces inflammatory cytokines in normal human lung fibroblasts (9). The mechanisms underlying BaP toxicity have not been fully elucidated. Nonetheless, it is considered that its ultimate metabolite, BaP-7,​8-diol-9,10-epoxide (BPDE), can form adducts with DNA, proteins, or lipids, leading to various BaP toxicities (10, 11).

BaP binds with high specificity to the aryl hydrocarbon receptor (AhR). Subsequently, AhR is translocated to the nucleus and binds to the AhR nuclear translocator, resulting in the increased expression of the Cytochrome P450 (CYP) metabolism genes, mainly CYP1A1 and B1. The inducible CYP enzymes metabolize BaP and generate the ultimate tumor-inducing forms (12, 13). Through CYP1A1-mediated metabolism of BaP by activating AhR, the BaP radical cation, free radical generation, and benzoquinones cause oxidative damage to biomolecules. The health effects of BaP are often explained in relation to BaP metabolism, generation of reactive oxygen species (ROS), and DNA damage induction. These mechanisms are critical to BaP-related toxicity, in addition to its primary genotoxicity (14, 15). However, a comprehensive understanding of the mechanisms underlying BaP-mediated pulmonary inflammatory diseases is lacking. In this study, we investigated the molecular mechanisms underlying BaP-inflamed lungs, which is critical for identifying and controlling the risks of inhalation exposure to BaP.

Inflammasomes are inducible multiprotein complexes that initiate inflammatory caspases and activate pro-inflammatory cytokines, interleukin (IL)-1β, and IL-18, which are essential events triggered by environmental irritants (such as microbes, exogenous crystals like silica, ultraviolet irradiation, and skin irritants) (16, 17). Recent studies have emphasized the relationship between air pollutant-induced toxicity and inflammasome activities as important mechanisms (18). The activation of inflammasomes by air pollutants is linked to the development of lung diseases (19). The inflammasome is composed of NOD-like protein (NLR), adaptor apoptosis-associated speck-like protein containing a caspase recruitment domain (ASC), and caspase-1, which is responsible for cleaving pro-IL-1β and pro-IL-18 proteins into their active forms. IL-1β is essential for inducting and developing inflammatory responses. Mature IL-1β secretion enhances inflammation and can lead to pathogenic responses and tissue injury. Two steps are required to activate the NLRP3 inflammasome in macrophages. The first step induces NLRP3 and pro-IL-1β protein expression *via* NF-kB- or MAP kinase-dependent pathway (priming step). The second step activates caspase-1 to generate IL-1β using various ligands (such as ATP, RNAs, or particulate matter) (20). In addition to NLRP3, NLRP1 is a key sensor mediating caspase-1 activation and cytokine maturation. The NLRP1 inflammasome is activated by lethal pathogen toxins and reduced intracellular ATP (21, 22). Furthermore, a recent study revealed that human NLRP1 is activated by enteroviral 3C protease during enteroviral infections (23). Exposure to ambient air pollutants also alters inflammasome activation. A previous report indicated that PM_2.5_-induced pulmonary inflammation is mediated by NLRP3 inflammasome activities, resulting in the pulmonary inflammation caused by PM_2.5_ exposure (24). The effects of BaP on inflammasomes in the lungs have also been studied. However, their contribution to inflammasome activity and inflammatory diseases in the lung remains uncertain. Moreover, it is essential to discover novel ligands that activate the inflammasome.

Various functional cells respond to BaP inhalation exposure (3). A comprehensive understanding of the pulmonary cells relevant to inhaled BaP is required. Lung epithelial cells have long been recognized as barriers to pathogens and play a role in gas exchange. The alveolar epithelium comprises two main cell types: alveolar type I (ATI) and alveolar type II (ATII). ATI cells cover most of the internal surface area of the lungs and mediate gas exchange. On the other hand, ATII cells are immune alveolar epithelium cells, as they possess immunomodulatory functions. They react with inhaled irritants, such as air pollutants, at the air-liquid interface and affect lung function and inflammation, cooperating with various immune cells. Inflammasomes in the lung epithelium have also been studied as a mechanism for pulmonary inflammation (19, 25). However, it remains unclear whether air pollutants, such as BaP, affect inflammasome activation in the lung epithelium. ATII cells synthesize lung surfactants, other proteins, and lipids with anti-inflammatory effects (26–28). Here, we focused on lung epithelial ATII cells, which initially interact with respirable irritants after inhalation, to provide insights into the molecular events initiating BaP-induced inflammation. In this study, we investigated the mechanism underlying BaP-induced inflammation by focusing on the inflammasome cascade in human lung epithelial A549 cells.

## 2 Materials and methods

### 2.1 PM_2.5_ sampling

The PM_2.5_ samples were collected using high-volume air samplers (SHIBATA HV-1000F; Shibata Scientific Technology, Tokyo, Japan) with a high-volume impactor for PM_2.5_ (HVI_2.5_) (Tokyo Dylec, Tokyo, Japan) at the Fukue (32.75°N, 128.68°E) monitoring site in Japan. PM2.5 sampling was performed according to previously described methods with some modifications. PM_2.5_ sampling was conducted at the Fukue monitoring site for 23 h between April 17 and 18, 2018. The island is on the western coast of Japan and is an outflow region for air pollutants in East Asia (29–31). The sampling air volume was 740 L/min. The PM_2.5_ samples were collected on polytetrafluoroethylene filters (WP-500-50, 8×10 inches; Sumitomo Denko Fine Polymer Co. Ltd., Osaka, Japan) after collecting PM_10_ on a glass fiber filter (Tokyo Dylec Co., Tokyo, Japan).

### 2.2 Cell culture and BaP treatment

A549 cells were cultured in Dulbecco’s modified eagle’s medium supplemented with 10% fetal bovine serum and 1% penicillin-streptomycin at 37°C with 5% CO_2_. PM_2.5_ suspension was applied to A549 cells at a final concentration of 2 µg/mL for four days. BaP (Tokyo Chemical Industry, Tokyo, Japan) and 6-Formylindolo[3,2-b] carbazole (FICZ, Sigma-Aldrich, St. Louis, MO) were dissolved in dimethyl sulfoxide (DMSO) and stored at 4 mM concentration. A549 cells were seeded into 6-well plates at 5×10^4^ cells concentration and treated with BaP or FICZ at the indicated concentrations and times. Control cells were treated with DMSO alone. For H_2_O_2_ stimulation, A549 cells were seeded into 6- or 12-well plates at 2×10^5^ or 5×10^5^ cells, respectively, and treated with 10 µM H_2_O_2_ (Nacalai Tesque, Tokyo, Japan) for 6 h. N-acetyl-l-cysteine (NAC, FUJIFILM Wako Chemicals, Tokyo, Japan) was reapplied to A549 cells at a final concentration of 1 mM for 1 h with 2 µM BaP.

### 2.3 Western blotting

BaP-treated cells were harvested using trypsin digestion and lysed with radioimmunoprecipitation (RIPA) assay lysis buffer (50 mM Tris-HCl, 150 mM NaCl, 0.1% SDS, 0.5% sodium deoxycholate, 1% NP-40, 1 mM Na_3_VO_4_, 5 mM sodium pyrophosphate, 50 mM NaF). The lysates were kept on ice for 10 min and centrifuged at 14,000 rpm for 10 min at 4°C. The supernatant protein concentration was measured using a BCA protein assay kit (Thermo Fisher Scientific, Waltham, MA). Equal amounts of protein were mixed with 4× reducing SDS-PAGE sample buffer (FUJIFILM Wako Chemicals) and heat-denatured for 5 min at 95°C. These proteins were separated on a 6%–15% gradient gel via SDS-PAGE and transferred to a nitrocellulose membrane. After blocking with Intercept Blocking Buffer (LI-COR, Lincoln, NE, USA) at room temperature for 30 min, primary antibodies were incubated overnight at 4°C, followed by incubation with appropriate fluorescent-conjugated secondary antibodies for 1 h at room temperature. After several washes, the fluorescence signals were detected using an Odessey scanner (LI-COR Biosciences, Lincoln, NE, USA). Monoclonal and polyclonal antibodies against NLRP1 (R&D Systems, Minneapolis, MN, USA), NLRP3 (Abclonal Biotechnology, Wuhan, China), AhR (BioLegend, San Diego, CA), ASC (Abclonal Biotechnology), pro-caspase-1, p20, p53 (R&D Systems), phosphorylated p53 (Ser 37, Cell Signaling, Beverly, MA, USA), actin, and vinculin were primary antibodies.

### 2.4 Quantitative RT-PCR

The cells were lysed using a TRIzol reagent (Invitrogen, Carlsbad, CA, USA). cDNA was synthesized using an RT Reagent Kit (Toyobo, Osaka, Japan). Real-time PCR (RT-PCR) was performed using SYBR Green Master Mix (Luna Universal qPCR master mix, New England Biolabs, Ipswich, MA) and analyzed using the MX3005P qPCR System (Stratagene, La Jolla, CA, USA). Gene expression was normalized to that of the internal control GAPDH gene, and the results were expressed as fold stimulation over the control. Primer sequences used are listed in **Supplemental Table 1**.

### 2.5 RNA interference

siRNAs were synthesized via Sigma-Aldrich. Naïve A549 cells were transiently transfected with 1 µM AhR siRNA (Sigma-Aldrich) or 5 µM TP53 siRNA (Sigma-Aldrich) by electroporation (CUY21EditII, BEX, Tokyo, Japan). MISSION siRNA Universal Negative Control #1 (Sigma-Aldrich) was used as the control siRNA. After 24 h of incubation, the cells were stimulated with 2 µM BaP and cultured for four days.

### 2.6 Flow cytometry

After BaP stimulation, the cells were stained with 10 µM 2′,7′-Dichlorofluorescin diacetate (DCFH-DA, Sigma-Aldrich) for 30 min at 37°C under 5% CO_2_. Stained cells were harvested via trypsin digestion. After washing several times, the cells were passed through a 0.45-μm nylon filter with. Flow cytometry was then performed on a FACSVerse flow cytometer (BD Biosciences, San Jose, CA). Representative histograms depicting the fluorescence intensity of DCFH-DA fluorescein-5-isothiocyanate (FITC) were presented, and the corresponding geometric mean fluorescence intensity (gMFI) was expressed in terms of fold change based on data acquired from independent experiments. The data was subsequently represented in a bar graph.

### 2.7 Immunostaining

BaP-stimulated cells were stained with wheat germ agglutinin (WGA; 10 µg/mL) for 5 min. The cells were fixed with 4% paraformaldehyde and permeabilized with 0.2% Triton X-100/PBS. Anti-NLRP3 antibody (A12694) (Abclonal Biotechnology, Wuhan, China), anti-NLRP3 antibody (Abcam, Cambridge, MA, USA), and rabbit IgG isotype control (negative control) were used for primary staining overnight at 4°C. Alexa Fluor 647 conjugated goat anti-rabbit IgG (Invitrogen) was used for the secondary staining at room temperature for 2 h. Stained cells were analyzed using a confocal laser scanning microscope (LSM-710, Carl Zeiss, Jena, Germany).

### 2.8 Immunoprecipitation

For ASC immunoprecipitation, the lysate supernatants were incubated with anti-ASC antibody (A1170) (ABclonal Biotechnology Wuhan, China) for 2 h at 4°C, followed by incubation with Dynabeads Protein-A (Life Technologies, Life Technologies, Grand Island, NY) for 1 h at 4°C. The beads were washed twice with lysis buffer and boiled in a sample buffer solution (containing 2-mercaptoethanol, FUJIFILM Wako Chemicals) for 5 min. For IL-1β detection in the culture supernatants, BaP-stimulated A549 cell supernatant was collected at each indicated time and incubated with anti-IL-1β antibody (R&D Systems) overnight at 4°C, followed by incubation with Dynabeads Protein-A (Life Technologies) for 3 h at 4°C. After washing, the magnetic beads were suspended in a sample buffer solution (containing 2-mercaptoethanol, FUJIFILM Wako Chemicals). Images were scanned using Odyssey (LI-COR Biosciences, Lincoln, NE, USA). Band intensity was measured using the ImageJ software (NIH, Bethesda, MD, USA). Each band intensity was normalized to that of the internal control, and the data are presented as a relative fold change. None of the images were modified using nonlinear adjustments.

### 2.9 Statistical analysis

Statistical significance was determined using GraphPad Prism version 7.0d (GraphPad Software, San Diego, USA). Statistical differences were determined using one-way analysis of variance (ANOVA) followed by Tukey’s or Dunnett’s multiple-comparison test, as appropriate (for comparing multiple groups). A *P*-value of <0.05 was considered significant.

## 3 Results

### 3.1 Air pollutants PM_2.5_ induces NLRP1 expression

**Figure 1:**
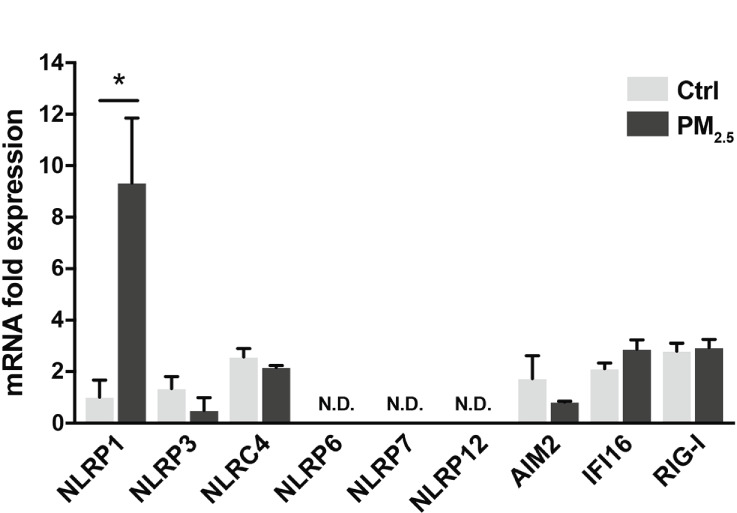
PM_2.5_ enhances NLRP1 expression in A549 cells. Expression of inflammasome-related PRRs in A549 cells. A549 cells were exposed to PM_2.5_ at 2 µg/mL for four days. PM_2.5_ was dissolved in deionized water to produce 20 µg/mL stock solutions, which were then added to the cell culture at the final concentration. Data are presented as the mean ± standard error. n = 3 independent experiments. *p<0.05; N.D, not detected. Control vehicle-treated control.

Inflammasomes induce respiratory inflammatory diseases by exposure to air pollutants, such as PM_2.5_. Pattern recognition receptors (PRRs) belonging to the NOD-like receptor (NLR) or the AIM2-like receptor family recognize pathogens and harmful signals. PM_2.5_ has induced pulmonary inflammation *via* the NLRP3 pathway (32). Here, we assessed the transcriptional changes in PRRs (NLRP1, NLRP3, NLRC4, NLRP6, NLRP7, NLRP12, AIM2, IFI16, and RIG-I) in A549 cells following response to PM_2.5_ exposure. The PM_2.5_ exposure significantly impacted NLRP1 expression in A549 cells (**Figure 1**). The other PRRs did not exhibit any marked differences (**Figure 1**).

### 3.2 BaP enhances NLRP1 expression in A549 cells

**Figure 2:**
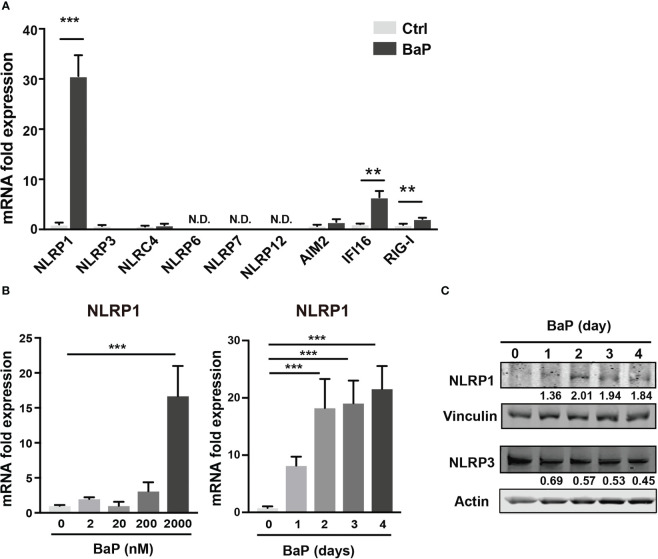
BaP enhances NLRP1 expression in A549 cells. BaP was dissolved in DMSO (final concentration, 0.05%) and added to A549 cells. **(A)** Expression of inflammasome-related PRRs in A549 cells. The cells were treated with 2 μM BaP for four days. **(B)** NLRP1 expression followed by BaP (0–2000 nM) for 0–4 days. **(C)** NLRP1 protein expression levels after four days of BaP treatment (2000 nM). Data are presented as the mean ± standard error. n = 4–9 independent experiments. **p<0.01, ***p<0.001; Control vehicle-treated control.

As mentioned above, BaP is a major component of PM_2.5_ and exhibits various biological functions. Here, we focused on the effects of BaP on PRR expressions in A549 cells. Annexin V and 7-AAD staining experiments revealed that cell viability did not differ with or without 2 µM BaP treatment for four days (**Supplementary Figure 1**). After four days of BaP treatment, NLRP1 expression was significantly enhanced in A549 cells (**Figure 2A**). We also observed a significant but less pronounced increase in IFI16 and RIG-I (**Figure 2A**). Furthermore, NLRP1 expression changes were dose- and time-dependent following BaP treatment (**Figure 2B**). The upregulated NLRP1 expression coincided with its elevated protein levels (**Figure 2C**), whereas a slight reduction in NLRP3 protein expression was observed during BaP treatment (**Figures 2A, C** **)**. In addition, the transcriptions of ASC, pro-caspase-1, and pre-formed IL-1β (pro-IL-1β), but not pre-formed IL-18 (pro-IL-18), were also markedly enhanced in response to BaP in a dose- and time-dependent manner (**Supplementary Figure 2**). These observations imply that BaP may significantly impact inflammasome signaling, which may be derived mainly from enhanced NLRP1 expression in lung epithelial cells.

### 3.3 BaP, but not FICZ, induced NLRP1 upregulation via AhR

**Figure 3:**
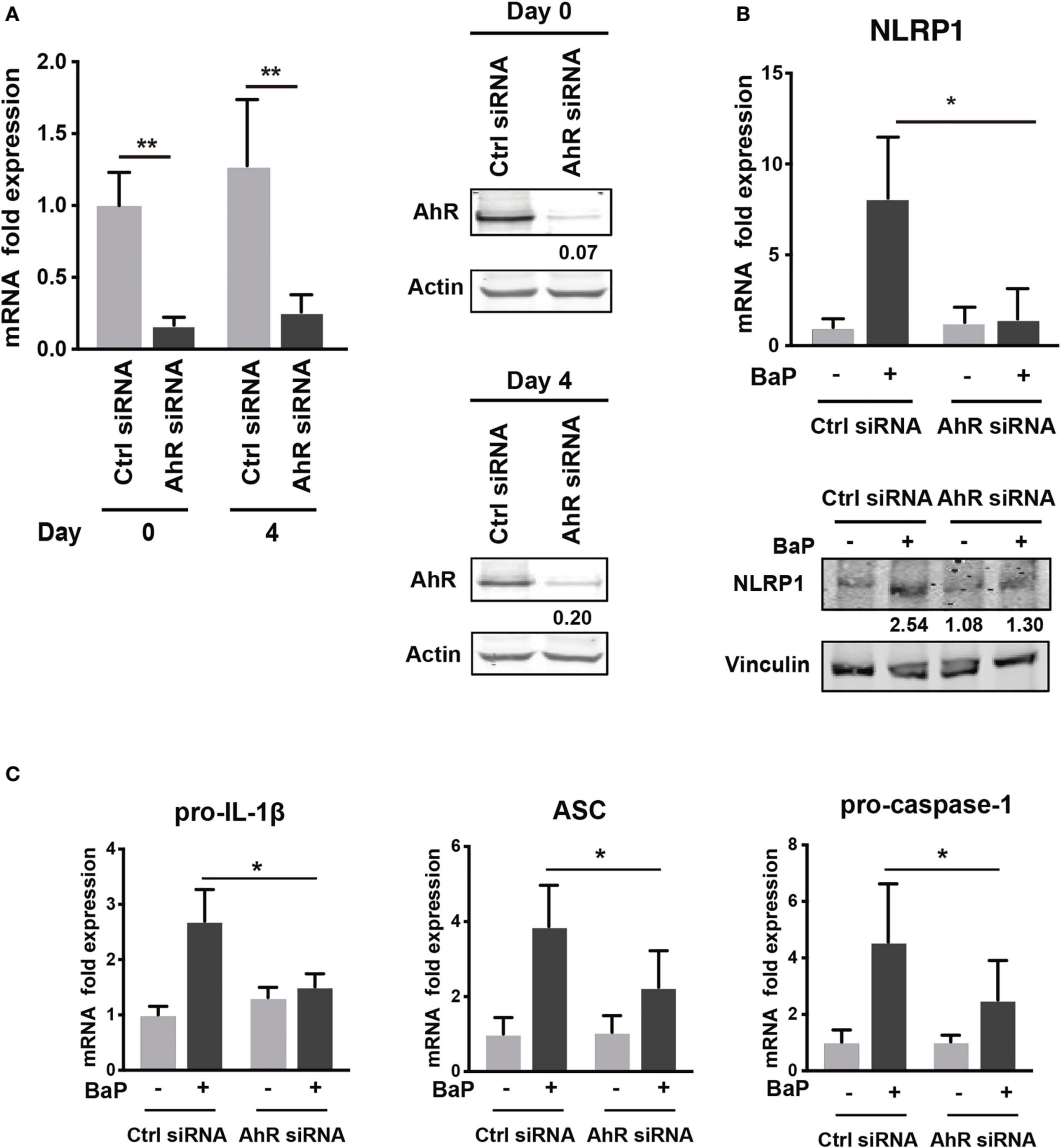
AhR knockdown by siRNA abrogates the BaP-induced NLRP1 expression. AhR participation in the enhanced NLRP1 expression in BaP-treated A549 cells was determined using AhR KD cells and an AhR antagonist. **(A)** AhR mRNA and protein expression in response to specific siRNAs. **(B)** NLRP1 expression after 2 µM BaP exposure for two (mRNA) or four (protein) days in AhR-knockdown cells and control cells. **(C)** Pro-IL-1β, ASC, and pro-Caspase-1 expressions after 2 µM BaP exposure for two days in AhR-knockdown cells and control cells. Data are presented as the mean ± standard error. n = 4–7 independent experiments; *p<0.05, **p<0.01.

**Figure 4:**
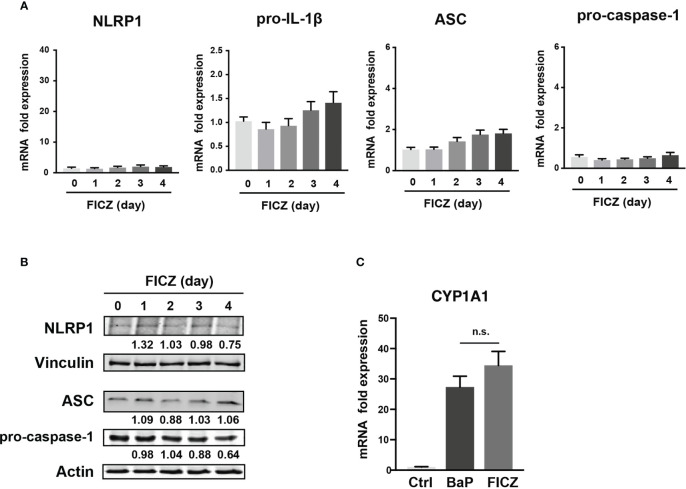
Effects of FICZ, a high-affinity ligand for AhR, on the NLRP1 expression in A549 cells. FICZ was dissolved in DMSO (final concentration, 0.05%) and added to A549 cells. NLRP-1, Pro-IL-1β, ASC, and pro-Caspase-1 expression at mRNA **(A)** and protein **(B)** levels. The cells were treated with 500 nM FICZ for 0–4 days. **(C)** CYP1A1 induction by BaP and FICZ treatment. Cells were treated with BaP (2 µM) and FICZ (500 nM) for four days. Data are presented as the mean ± standard error. n = 3–9 independent experiments. n.s., not significant.

To examine the mechanism by which BaP enhances NLRP1 expression in A549 cells and its subsequent consequences, we first assessed BaP-induced toxicity mediated by AhR activation (33). In this study, we utilized a knockdown approach using siRNA specific for AhR. We confirmed AhR knockdown at the mRNA and protein levels after siRNA transfection (**Figure 3A**), which was maintained for up to four days (**Figure 3A**). Day 0 was set as 24 h after siRNA transfection when the cells were exposed to BaP in the experiments illustrated in **Figures 3B, C**. AhR knockdown in A549 cells significantly abrogated BaP-induced NLRP1 mRNA and protein expression (**Figure 3B**). In addition to AhR knockdown in A549 cells, pro-IL-1β, ASC, and pro-caspase-1 were significantly abrogated (**Figure 3C**). These data indicated that AhR is involved in the upregulation of NLRP1 transcription by BaP. AhR directly or indirectly regulates inflammasome-related gene expression; therefore, we compared the effects of BaP or FICZ, high-affinity ligands for AhR, on NLRP1 expression in A549 cells. In contrast to the results of BaP presented in **Figure 2**, FICZ treatment did not enhance NLRP1, ASC, and pro-caspase-1 expressions at the mRNA and protein levels (**Figures 4A, B** **)**, although FICZ sufficiently induced CYP1A1 expression at a BaP equivalent level (**Figure 4C**). These data indicate functional differences between BaP and FICZ in AhR activation and subsequent NLRP1 expression in A549 cells.

### 3.4 ROS production is involved in the BaP/AhR-mediated NLRP1expression

**Figure 5:**
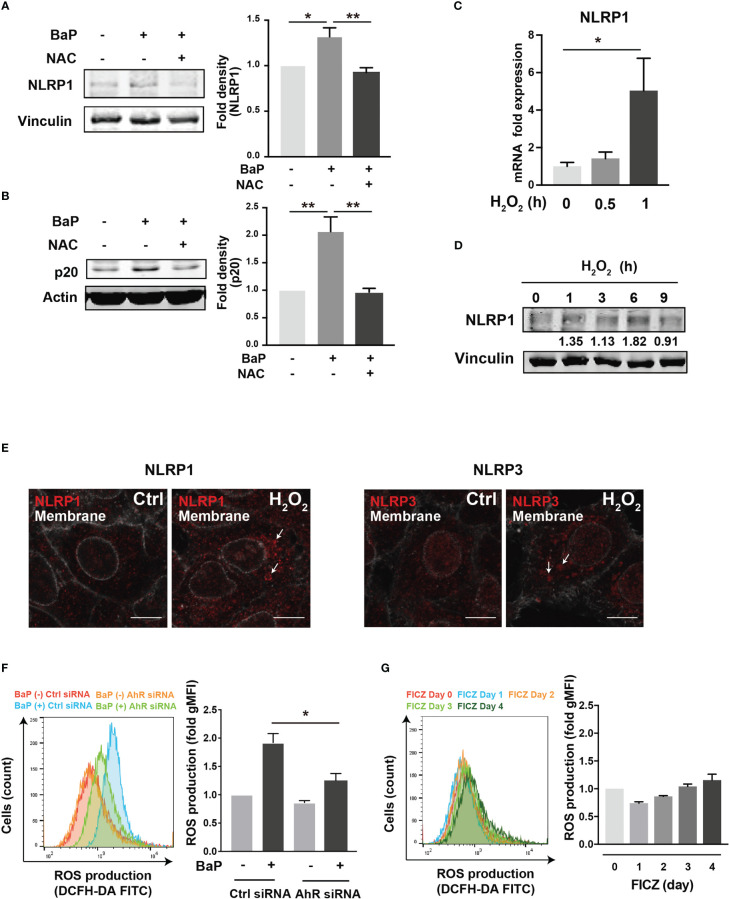
ROS generation is involved in the BaP-induced NLRP1 expression. The involvement of ROS was measured in BaP-treated A549 cells using NAC as an antioxidant. **(A, B)** NAC was added to the cells at a concentration of 5 μM 1 h prior to BaP application. Abrogated BaP-induced NLRP1 and p20 expression after NAC treatment were analyzed. Densitometry analysis of the western blot data of NLRP1 and p20 is shown in the bar graphs. Expression of NLRP1 mRNA **(C)** and protein **(D)** under H_2_O_2_ treatment (1 mM). **(E)** Analysis of components of the inflammasome protein complex in the H_2_O_2_-treated A549. The cells were then exposed to H_2_O_2_ at 1 mM for 9 h. Representative images (scale bar = 10 μm) of confocal microscopic observations of H_2_O_2_-treated cells. **(F, G)** AhR KD and control cells were exposed to BaP **(F)** at 2 µM and FICZ **(G)** at 500 nM for four days. The induced ROS were analyzed using flow cytometry, and the fold production of ROS was represented as the mean fluorescence intensity of DCF. Data are presented as the mean ± standard error. n = 3–4 independent experiments. *p<0.05, **p<0.01; NAC, N-acetyl-l-cysteine.; gMFI, geometric mean fluorescence intensity; DCF, dichlorofluorescein.

To further investigate the downstream of the BaP/AhR signal transduction pathway and the cause of FICZ-induced NLRP1 expression failure, we assessed reactive oxygen species (ROS) generation. Excessive ROS production by BaP disturbs redox homeostasis, causing oxidative stress, which results in increased cytotoxicity and inflammatory responses, including inflammasome activation. Indeed, ROS activates NLRP3 inflammasome (34). Furthermore, a recent study demonstrated that NOX2-induced ROS accumulation activates NLRP1 inflammasome (35). However, it is not fully understood whether BaP-induced ROS generation leads to enhanced pulmonary inflammasome activity in lung epithelial cells damaged by exposure to components of PM_2.5_ Therefore, we examined the relationship between BaP-induced ROS production and NLRP1 up-regulation in A549 cells. ROS production was analyzed using DCFH-DA fluorescence, which revealed a significant increase based on BaP treatment (**Supplementary Figures 3A, B**). When ROS generation was inhibited by pretreatment with the ROS scavenger—antioxidant NAC—(**Supplementary Figure 3C**), BaP-induced NLRP1 upregulation and possibly subsequent induction of active caspase p20 were attenuated (**Figures 5A, B** **)**. Furthermore, we confirmed the involvement of ROS in NLRP1 expression in A549 cells using H_2_O_2_ as an oxidant. As expected, H_2_O_2_-induced ROS production (**Supplementary Figure 3D**) effectively increased NLRP1 expression at the mRNA and protein levels (**Figures 5C, D** **)**. When we observed NLR aggregation, which suggests inflammasome formation, H_2_O_2_-treated A549 cells had aggregated NLRP1 and NLRP3 (**Figure 5E**), indicating H_2_O_2_-derived inflammasome activation. BaP-induced ROS production was significantly reduced in AhR-KD cells (**Figure 5F**). FICZ could activate caspase p20 (**Supplementary Figure 4**); however, it did not induce ROS production (**Figure 5G**). These data are consistent with those of previous reports (36, 37). A difference in ROS generation potency between both AhR ligands (BaP and FICZ) seems to exist in A549 cells. These observations, combined with the results in **Figures 2** **-** **4**, collectively indicate that the AhR-ROS axis might be involved in BaP-induced NLRP1 expression in A549 cells.

### 3.5 p53 regulates BaP-induced NLRP1 increase

**Figure 6:**
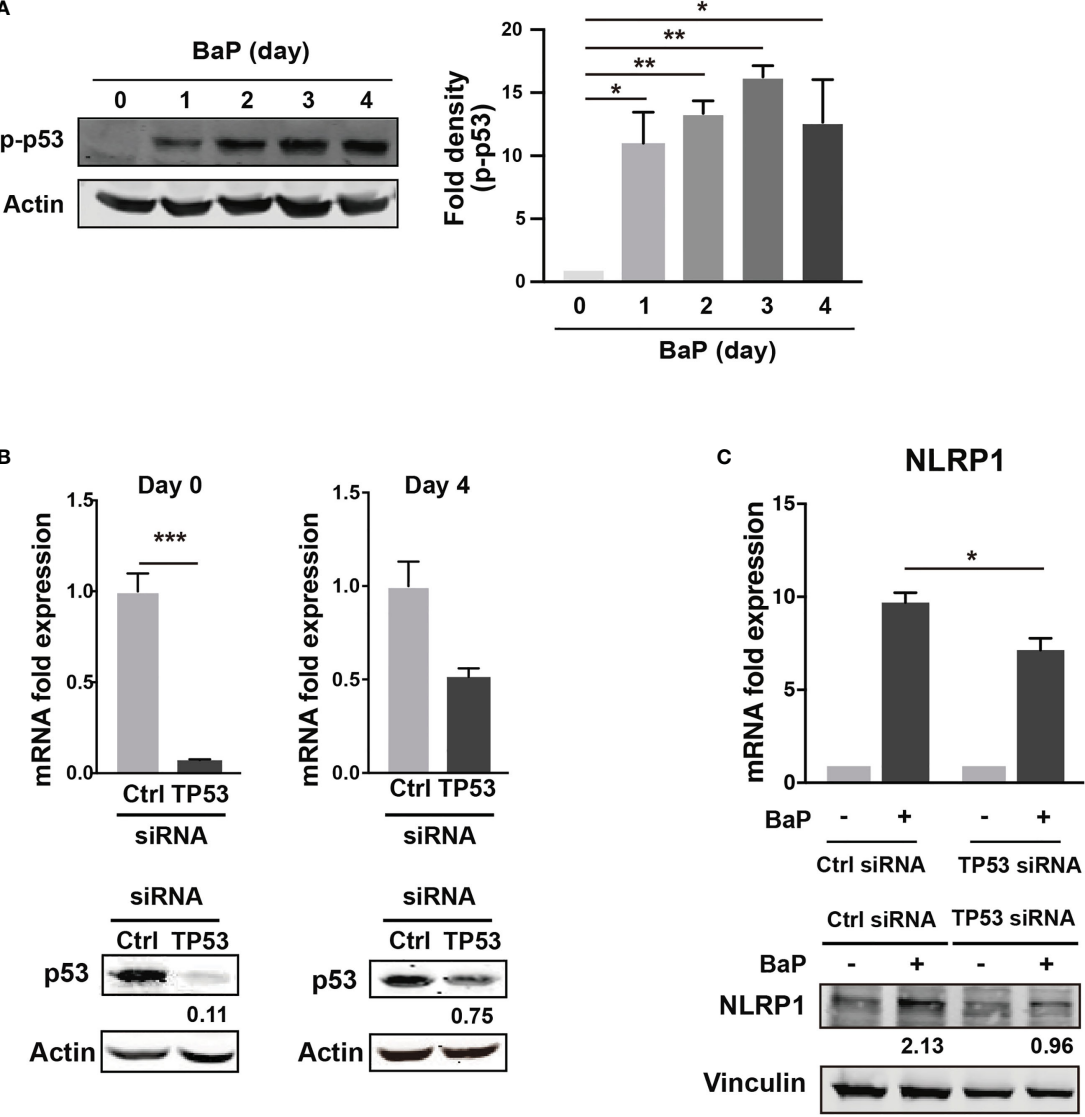
Determination of the role of p53 in the BaP-induced NLRP1 expression. When p53 was knocked down using TP53-siRNA in A549 cells, BaP-induced NLRP1 expression was attenuated. **(A)** Phosphorylation of p53 in BaP-treated cells. Densitometry analysis of the western blot data of p53 phosphorylation is shown in the bar graph. **(B)** Expression levels of p53 in response to specific siRNAs. **(C)** Expression of NLRP1 mRNA and protein in p53 knockdown and control cells. BaP (2 µM) was added and incubated for four days. Data are presented as the mean ± standard error. n = 3–4 independent experiments. *p<0.05, **p<0.01, ***p<0.0001.

There is a link between BaP-induced ROS generation and NLRP1 gene upregulation. To investigate this link, we hypothesized that the p53 function is compromised by the mechanisms presented in this study (38, 39). The tumor suppressor protein—p53—is a key transcription factor that regulates the expression of genes involved in apoptosis and cell cycle arrest. Tebaldi et al. identified a putative p53 responsive element approximately 700 bp upstream of the transcription start site of NLRP1 (40). NLRP1 expression is regulated by p53 (39). It is generally accepted that the p53 pathway can be activated by BaP exposure via genotoxic and non-genotoxic pathways, including ROS generation (41, 42). We also observed that p53 phosphorylation was markedly enhanced by BaP treatment in A549 cells (**Figure 6A**). Next, we tested whether p53 is involved in NLRP1 upregulation by BaP in cells. The p53 expression was knocked down by siRNA against TP53, and we obtained approximately 90% (day 0) and 25% (day 4) knockdown efficiencies following siRNA transfection (**Figure 6B**). Day 0 was set as 24 h after siRNA transfection when the cells were exposed to BaP in the experiments illustrated in **Figure 6C**. When BaP was applied to TP53 KD cells, BaP-induced NLRP1 upregulation was significantly inhibited (**Figure 6C**), indicating that p53 plays an essential role in the BaP-induced NLRP1 upregulation in A549 cells. Previous studies have reported that excessive ROS generation can increase p53 levels (38, 43, 44). Similarly, we also observed that NAC treatment resulted in the inhibition of BaP-induced p53 activation, verifying that the ROS/p53 axis is involved in BaP-induced p53 activation in A549 cells (**Supplementary Figure 5**). These data, combined with previous publications, suggest that the BaP-induced p53 upregulation could be at least partially explained by ROS generation but may also be due to its direct regulation by the enzymatic conversion of BaP and its metabolites.

### 3.6 BaP differentially regulates NLRP1 and NLRP3 expression and alters inflammasome complex for IL-1β secretion

**Figure 7:**
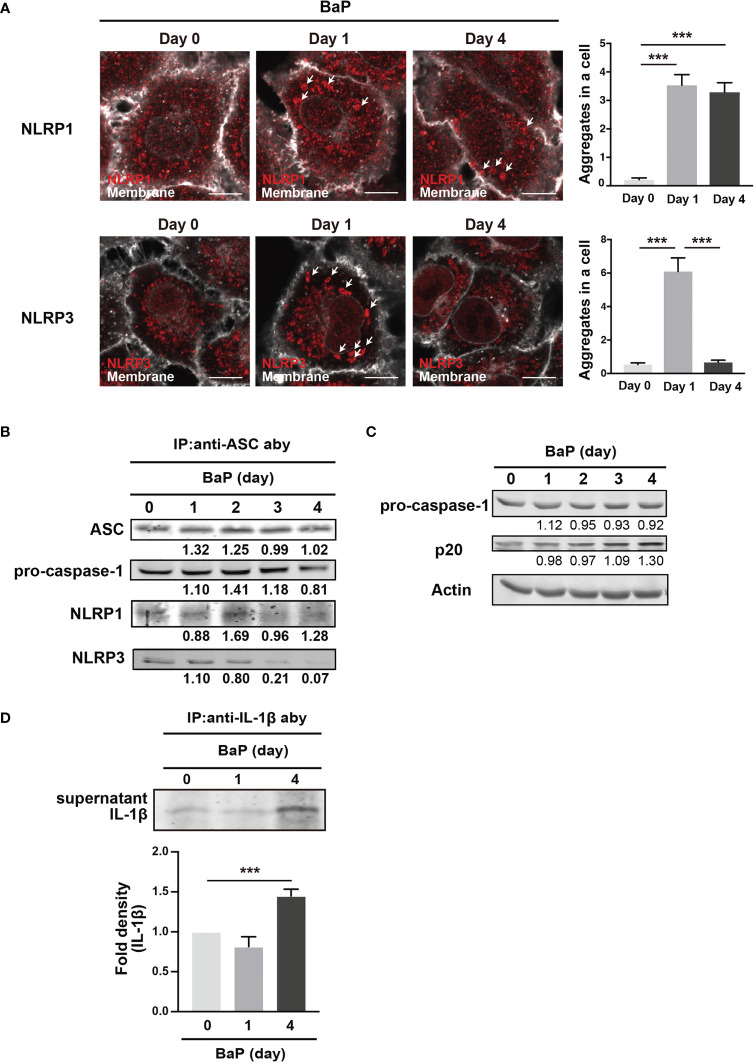
Detection of inflammasome formation using co-immunoprecipitation assay. Analysis of inflammasome protein complex components in BaP-treated A549 cells. Cells were exposed to 2 µM BaP for four days. **(A)** Representative images (scale bar = 10 μm) and quantitative bar graphs of confocal microscopy observations of BaP-treated cells. **(B)** Representative immunoblot of the co-immunoprecipitation assay using an anti-ASC antibody. **(C)** time-dependent increase in p20 expression in response to BaP treatment. Densitometry analysis of the western blot data of IL-1β is shown in the bar graph. **(D)** IL-1β production in BaP-treated cells. Data are presented as the mean ± standard error. n = 3–6 independent experiments. ***p<0.001.

**Figure 8:**
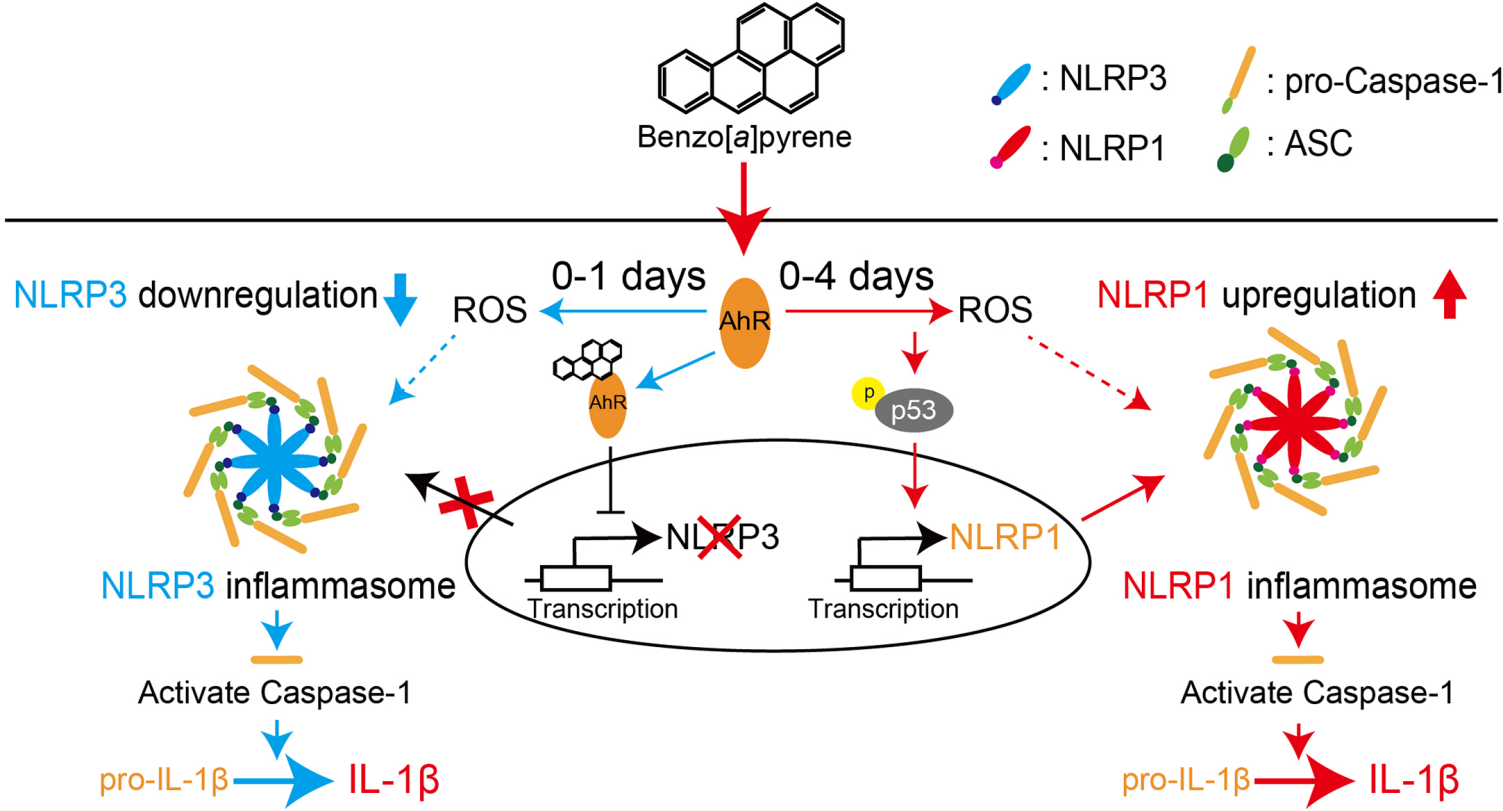
Proposed scheme for regulating NLR proteins by BaP in the lung epithelial cells. We demonstrated that BaP enhances NLRP1 expression in lung epithelial A549 cells and leads to activation of the NLRP1 inflammasome cascade in the cells. At the same time, BaP also regulates the NLRP3 inflammasome through an independent pathway. There is a time gap between NLRP1 and the NLRP3 inflammasome caused by BaP.

We demonstrated the significant BaP-induced upregulation of NLRP1 expression in A549 cells and its canonical mechanisms. In this study, we also observed a reduction in NLRP3 expression in response to BaP, as illustrated in **Figure 2**. These observations led to our focus on inflammasome protein complex formation. Confocal microscopic images of the BaP-treated cells revealed that the BaP-induced NLRP1 inflammasome was sustained during the observation. In contrast, the NLRP3 inflammasome was transiently observed (**Figure 7A**). This time gap was similar to that of the protein expression patterns (**Figure 2C**). Given the significantly enhanced inflammasome formation, we analyzed inflammasome complexes using a co-immunoprecipitation assay with an anti-ASC antibody. NLRP1 was co-immunoprecipitated with ASC during the four observation days. In contrast, co-immunoprecipitation of NLRP3 was not observed on day 3 (**Figure 7B**). Thus, BaP appears to affect NLRP1 and NLRP3 inflammasome activation in A549 cells, in which there may be a shift in the intracellular inflammasome cascade from the NLRP3 to the NLRP1 dominant pattern. To confirm the shift in the inflammasome complex from NLRP3 to NLRP1 on caspase-1 activity, we examined active caspase p20 in BaP-treated A549 cells. As illustrated in **Figure 7C**, a time-dependent increase in p20 was observed. Finally, we investigated the consequence of this shift in inflammasome formation by BaP and evaluated the secretion of IL-1β from the cells. The detection of extracellular IL-1β protein resulted in a significant increase in its secretion in BaP-treated cells (**Figure 7D**). This suggests that the NLRP1 dominant inflammasome formation pattern could induce IL-1β production and secretion, which could be deemed NLRP1 inflammasome activation (**Figure 8**).

## 4 Discussion

We demonstrated that BaP enhanced NLRP1 expression and activated the NLRP1 inflammasome cascade in lung epithelial A549 cells. In addition, BaP downregulated NLRP3 inflammasome through different pathways with NLRP1. BaP altered the dominant NLR protein in the inflammasome from NLRP3 to NLRP1 by enhancing NLRP1 expression, which may be involved in prolonged inflammation.

PM_2.5_ affects the NLRP3 inflammasome pathway (45). Here, we observed BaP and PM_2.5_ serve as novel regulators of the NLRP1 inflammasome (**Figures 1**, **2**). AhR is a conserved environmental sensor that responds to various xenobiotic exposures. BaP binds with high affinity to AhR and activates the downstream signaling pathways. In this study, we observed that BaP enhances NLRP1 in A549 cells. The significant BaP-induced *NLRP1* upregulation in A549 cells was mediated through AhR (**Figures 2**, **3**). AhR directly or indirectly regulates the expression of several target genes and interacts with other transcriptional regulators (33, 46). To our knowledge, no study has described that the NLR gene is under the control of an AhR-responsive promoter element, suggesting that an indirect pathway might be involved in the BaP-AhR-NLRP1 axis in A549 cells. Therefore, we focused on ROS production in the cells. ROS are well-known mediators of the indirect pathway that modulates AhR signaling. Intracellular ROS levels in A549 were markedly enhanced by BaP stimulation (**Supplementary Figure 3**), and ROS enhancement might be involved in the NLRP1 upregulation (**Figure 5**). In detecting the ROS-mediated pathway in BaP-induced NLRP1 expression, FICZ, a high-affinity AhR ligand, did not demonstrate efficacy equivalent to BaP, suggesting an indirect regulation of *NLRP*1 through AhR (**Figure 4**). Additionally, the extent of ROS production under FICZ treatment did not mimic that observed under BaP treatment (**Figure 5**, **Supplementary Figure 3**). These paradoxical data may be explained by their potential as ligands for AhR. However, both have been recognized as high-affinity AhR ligands. However, FICZ inhibits AhR-induced ROS production, potentially through Nrf2 activity (36, 37), whereas BaP strongly induces ROS (47). Their functions have been discussed and are yet to be partially clarified (48). Our findings and previous reports reveal that BaP enhanced *NLRP1* in A549 through AhR/ROS-mediated mechanisms.

p53, a transcription factor with a tumor suppressor function, can regulate *NLRP1* expression (38, 39). BaP highly phosphorylated p53 (**Figure 6**), and TP53 KD cells revealed that BaP-induced p53 activation was likely involved in *NLRP1* expression enhancement (**Figure 6**). p53 functions as a transcription factor and can trigger various anti-proliferative programs by activating or repressing key effector genes (49, 50). In addition, our studies using NAC and H_2_O_2_ revealed that NAC treatment inhibited BaP-induced p53 activation by inhibiting ROS production (**Supplementary Figures 3**, **5**). This consistently suggests that the ROS/p53 axis is involved in NLRP1 enhancement via p53 activation in BaP-treated A549 cells. Collectively, p53 seems involved in AhR/ROS-mediated *NLRP1* up-regulation in A549 cells.

We conducted co-immunoprecipitation using an anti-ASC antibody to confirm inflammasome activation in A549 cells stimulated with BaP. NLRs sense and are activated by intracellular changes caused by extracellular molecules. After activation, NLRs recruit the adaptor molecule—ASC, which binds to pro-caspase-1, leading to autocatalytic processing and activation. NLRP1 activation is uniquely dependent on proteasomal degradation (51). Pathogen-induced proteasome-mediated degradation of the amino-terminal domains of NLRP1 releases the carboxyl-terminal fragment, sufficient to recruit caspase-1 and activate the inflammasome. **Figure 7** illustrates that caspase-1 activation, as indicated by blotting for p20, was enhanced by BaP treatment, indicating that BaP-induced NLRP1 expression leads to NLRP1 inflammasome formation. Furthermore, the co-immunoprecipitation assay confirmed that BaP differentially regulated NLRP1 and NLRP3 during the experiments for up to four days. These results supported our speculation raised in **Figure 2**. BaP appears to differentially regulate NLRP1/NLRP3 inflammasome in lung epithelium, suggesting a sustained inflammatory state within the lung in response to ambient air pollution exposure.

We investigated the IL-1β secretion induced by inflammasome formation. Our results revealed increased extracellular IL-1β levels on day 4 of BaP stimulation. IL-1β has a short plasma half-life (52); therefore, the promoted secretion may not simply be explained by the accumulation during the four days of BaP stimulation, which may be associated with the altered formation of BaP-induced inflammasomes in A549 cells. IL-1β is a cytokine that strongly induces inflammation and acts on various immune cells to induce inflammatory responses. Stimulating the IL-1 receptor by IL-1β activates crucial inflammatory signaling pathways, such as NF-κB and MAPK, and subsequent cytokine expression (52). The provoked secretion of IL-1β by type II alveolar epithelial cells further enhances the inflammatory response of nearby alveolar immune cells migrating due to inflammation, which may exacerbate lung inflammation. In the context of macrophage activation in response to BaP, PAHs containing BaP suppress the differentiation of monocytes into macrophages (53), which may affect the number of macrophages at the inflamed site. In addition, alveolar macrophages that phagocytose exogenous particles can leave the alveoli by migrating toward the bronchiolar region to eliminate particles. Thus, while the activities of pulmonary macrophages in BaP-induced inflammation have been studied extensively, the role of pulmonary epithelial cells in local inflammation remains relatively unexplored. Our findings suggest that type II alveolar epithelial cells may be involved in sustained immune responses toward BaP in the lung through NLRP1 and NLRP3 inflammasome activation. In the context of the A549 cell inflammasome response to BaP exposure, our study revealed a narrow concentration range wherein expression of PRR genes was abruptly increased by BaP concentrations of 200–2000 nM. A previous study has revealed a similar pattern with regard to the impact of PM_2.5_ on human bronchial epithelial cells (54). However, the impact of PM_2.5_ on human health is not exclusively attributable to BaP, suggesting that the optimal concentration of BaP may be limited. In addition, BaP is metabolized by CYP enzymes resulting in a number of metabolites being formed. At low concentrations, the enzymes may exhibit suboptimal metabolic efficiency towards BaP, thereby conferring effectiveness only within a restricted concentration range. Therefore, the precise threshold concentration of BaP for relevant cells is a crucial issue to address in a future investigation.

The NLRP3/caspase-1 pathway is crucial to PM_2.5_-induced pneumonia (24). Therefore, the relationship between PM_2.5_-induced inflammation and the activation mechanisms of the inflammasome pathway is essential. Further experiments are needed to explain why the NLRP1 inflammasome cascade in A549 cells is activated by BaP stimulation. However, this study revealed that BaP, a novel inducer of NLRP1 in A549 cells, is associated with the NLRP1 inflammasome cascade activation. The extent of NLRP1 induction in A549 cells was more significant than that of NLRP3 suppression. Therefore, we suggest that NLRP1 activation is the dominant mechanism against BaP in the lung epithelium.

It is commonly accepted that airborne irritants affect NLRP3 inflammasome and enhance pulmonary inflammation. However, our findings suggest that BaP can switch inflammasome cascades from the NLRR3 inflammasome to the NLRP1 inflammasome, implying a mechanism for sustained lung inflammation. Consequently, we conclude that BaP enhances NLRP1 expression in lung epithelial A549 cells and leads to activation of the NLRP1 inflammasome cascade in these cells, while BaP also seems to regulate the NLRP3 inflammasome through an independent pathway. There is a time gap between NLRP1 and the NLRP3 inflammasome caused by BaP. Our findings may contribute to a novel understanding and strategy to control lung inflammation caused by environmental irritants.

## Data availability statement

The original contributions presented in the study are included in the article/**Supplementary Material**. Further inquiries can be directed to the corresponding author.

## Author contributions

RK, YN, TI, CA, YI, TS, and RS contributed by conducting experiments, summarizing data, and generating the necessary reagents. RS conceived of and directed the study. RK, YN, and RS participated in the writing of the manuscript. All authors contributed to the article and approved the submitted version.
