# Supplementary material for: Benzo[a]pyrene induces NLRP1 expression and promotes prolonged inflammasome signaling
Source: Front Immunol. 2023 May 4;14:1154857. doi: 10.3389/fimmu.2023.1154857 (PMC10192748; doi:10.3389/fimmu.2023.1154857)
Supplement: Supplementary file 1 [file DataSheet_1.docx]

Supplementary Material

1. **Supplementary Tables**

**Supplementary Table 1.** Sequences of primers used for gene expression analysis using qPCR.

| Gene | Forward | Reverse |
| --- | --- | --- |
| human NLRP1 | GCTGGACCAGACAACTCTGA | GGTTTCCGTCTGCTGAAGAT |
| human NLRP3 | GATCTTCGCTGCGATCAACA | GGGATTCGAAACACGTGCATTA |
| human NLRC4 | TGAACTGATCGACAGGATGAAC | GTCTCCAGTTTTTCAACCCAAG |
| human NLRP6 | ACTGTGCCATCTGAGCAGCCTC | TCACTGAGCCTGTTGTGGAGGA |
| human NLRP7 | GGCCAGAATCATTTGTGGAA | TTTTCTTTCACTTCCTCCAACA |
| human NLRP12 | AGCACAGAAGCCATCTCCTGAC | AGCACAGAAGCCATCTCCTGAC |
| human AIM2 | CACCAAAAGTCTCTCCTCATGTT | AAACCCTTCTCTGATAGATTCCTG |
| human IFI16 | GATGCCTCCATCAACACCAAGC | CTGTTGCGTTCAGCACCATCAC |
| human RIG-I | GGACGTGGCAAAACAAATCAG | GCAATGTCAATGCCTTCATCA |
| human ASC | ATCCAGGCCCCTCCTCAGT | GTTTGTGACCCTCGCGATAAG |
| human pro-caspase-1 | GCCTGTTCCTGTGATGTGGAG | TGCCCACAGACATTCATACAGTTTC |
| human AhR | GTCGTCTAAGGTGTCTGCTGGA | CGCAAACAAAGCCAACTGAGGTG |
| human p53 | GCCCAACAACACCAGCTCCT | CCTGGGCATCCTTGAGTTCC |
| human GAPDH | CATGTTCGTCATGGGTGTGAACCA | ATGGCATGGACTGTGGTCATGAGT |

1. **Supplementary Figures**

**
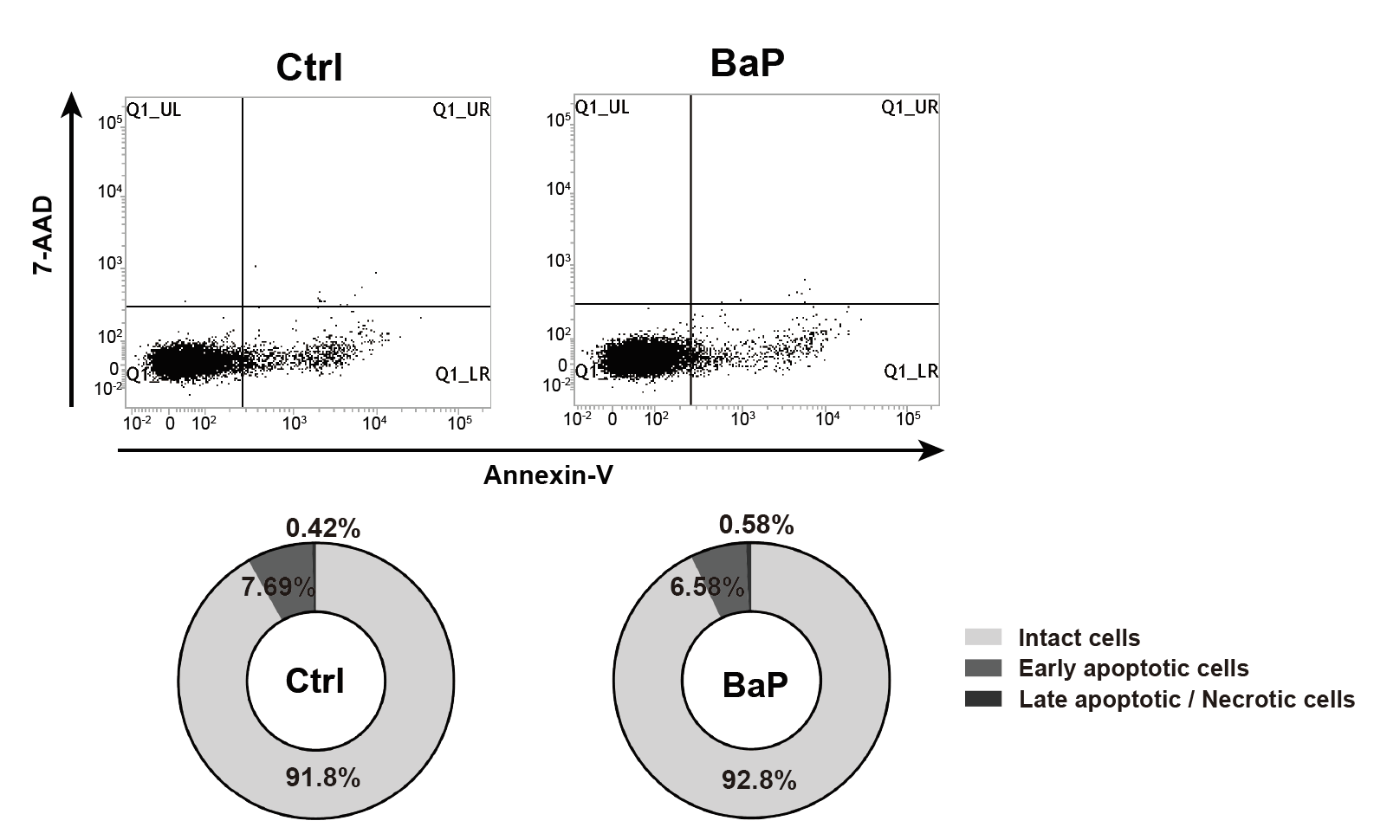
**

**Supplementary Figure 1**

**Determination of BaP cytotoxicity in A549 cells**

The viability of BaP-treated cells was assessed with Annexin V and 7-AAD staining. Cells were exposed to 2 µM BaP for four days. Early apoptotic cells were Annexin V-positive and 7-AAD-negative, whereas late apoptotic cells were Annexin V/7-AAD-double-positive. n = 3 independent experiments.

**
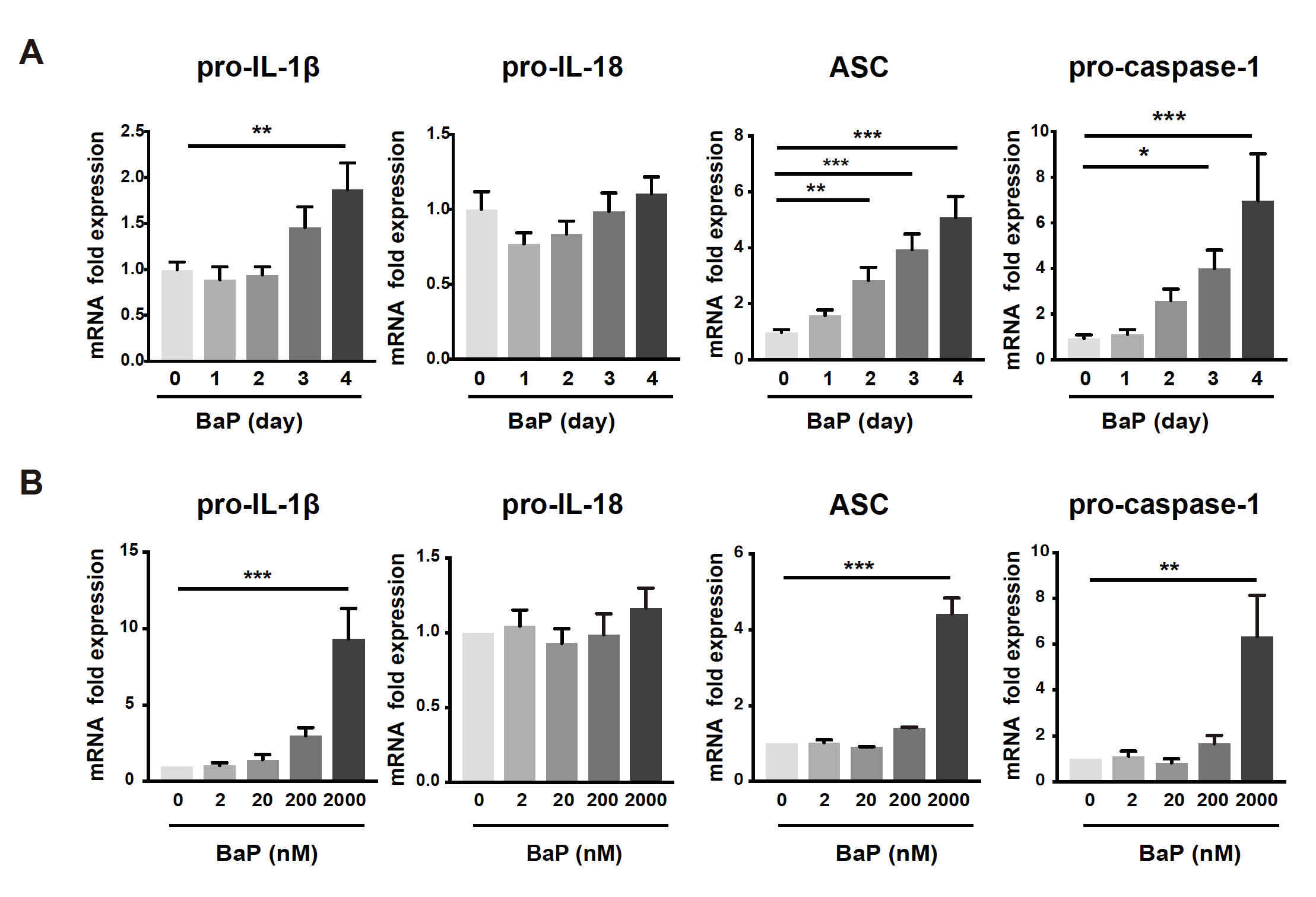
**

**Supplementary Figure 2**

**BaP-induced expression of inactivate precursor of cytokines and adaptor molecules**

**(A)** Time- and (**B)** dose-dependent expression of pro-IL-1β, pro-IL18, ASC, and pro-caspase-1 was assessed in BaP-treated A549 cells. Data are presented as the mean ± standard error. n = 3–9 independent experiments. *p<0.05, **p<0.01, ***p<0.001.

**
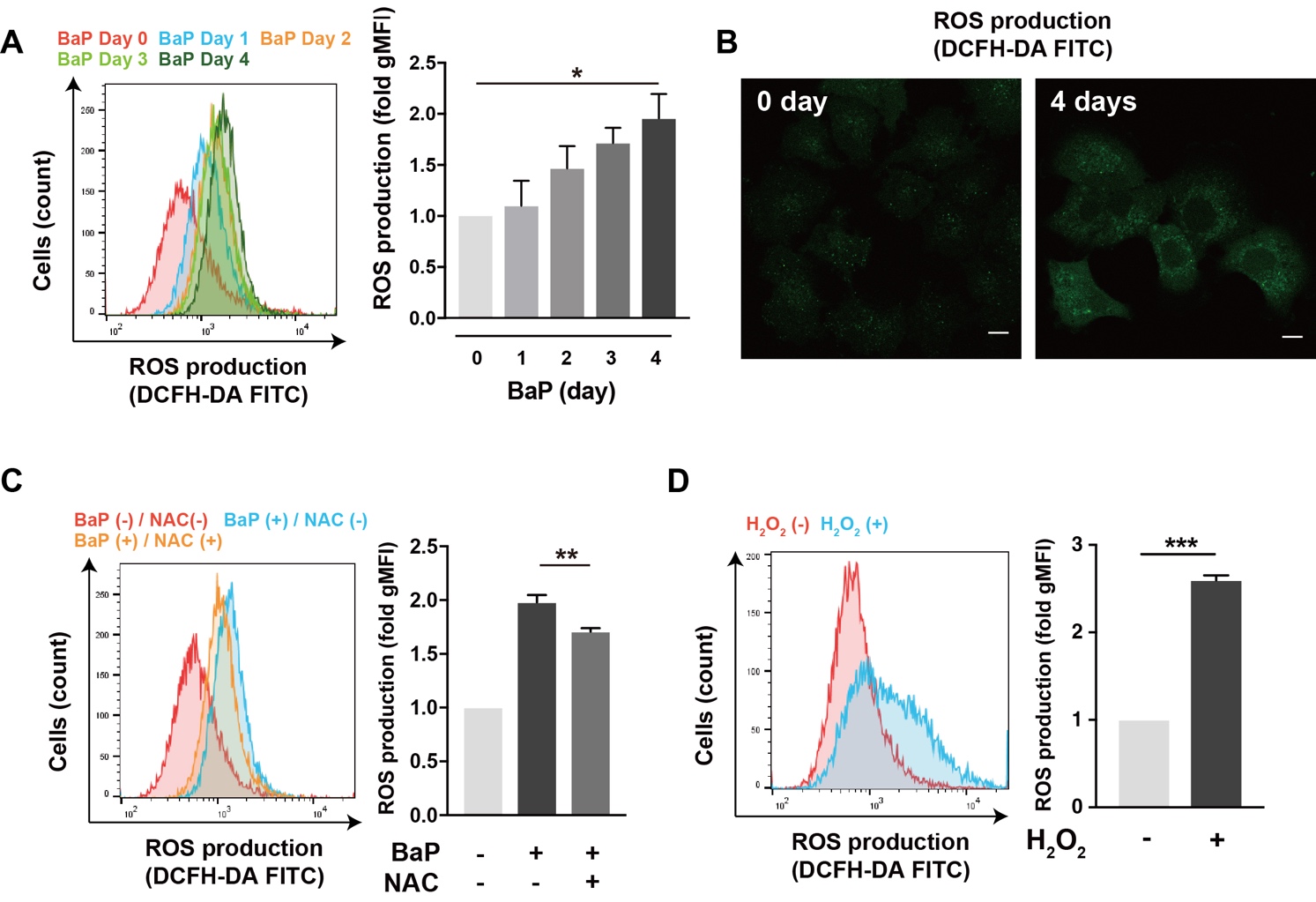
**

**Supplementary Figure 3**

**Detection of the BaP-induced ROS production in A549 cells**

ROS production in A549 cells was analyzed using DCF fluorescence. A549 cells were treated with 2µM BaP. **(A)** Flow cytometry analysis of ROS levels. Representative flow histograms and quantitative bar graphs of DCF fluorescence. **(B)** Relative microscopic images (scale bar = 10 μm) obtained using confocal laser scanning microscopy. **(C)** Inhibition of ROS production by NAC treatment in A549 cells. Flow cytometry analysis of ROS. Representative flow histograms and quantitative bar graphs of DCF fluorescence. **(D)** Induction of ROS production by H_2_O_2_ treatment (1 mM). Flow cytometric analysis of ROS. Representative flow histograms and quantitative bar graphs of DCF fluorescence. *p<0.05, **p<0.01, ***p<0.001. gMFI, geometric mean fluorescence intensity; DCF, dichlorofluorescein.


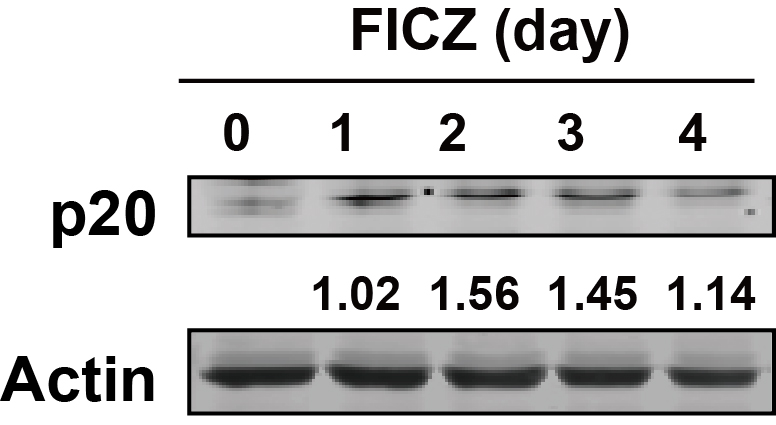


**Supplementary Figure 4**

**Activation of p20 in the FICZ-treated A549 cells**

The cells were treated with 500 nM FICZ for four days. Cleaved caspase-1, p20, was analyzed using the western blot. Representative blots are presented from three independent experiments.

**
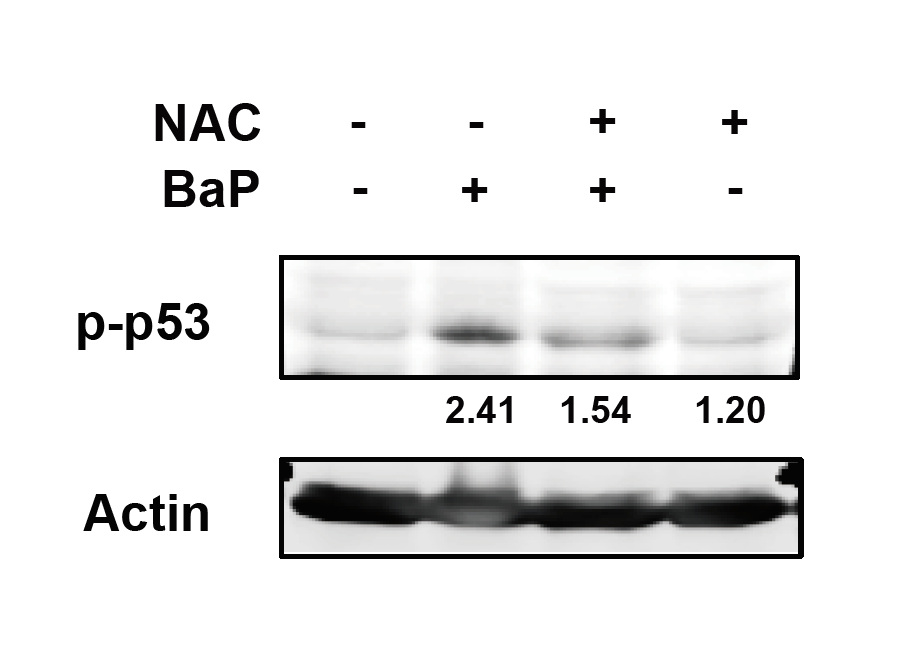
**

**Supplementary Figure 5**

**Inhibition of the BaP-induced p53 phosphorylation by NAC treatment**

NAC was treated in the A549 cells at 1 μM before 3 h of BaP application. BaP was added at a concentration of 2 µM and incubated for four days. Phosphorylation of p53 was analyzed using western blotting. Representative blots are presented from four independent experiments.
